# Editorial for the Special Issue “Challenges of Biofilm-Associated Bone and Joint Infections”

**DOI:** 10.3390/microorganisms14061276

**Published:** 2026-06-05

**Authors:** Jaime Esteban, Christof Berberich

**Affiliations:** 1 Department of Clinical Microbiology, IIS-Fundacion Jimenez Diaz, Universidad Autonoma de Madrid, CIBERINFEC-CIBER de Enfermedades Infecciosas, 28040 Madrid, Spain; 2 Department of Medical Affairs, Heraeus Medical GmbH, 61273 Wehrheim, Germany; christof.berberich@heraeus.com

Prosthetic joint infections (PJI), although a relatively uncommon event in orthopedic surgery, are a devastating problem that implies high morbidity, mortality, and economic costs for the Healthcare System [1]. Proper management of these patients should include the participation of different medical and surgical specialists, because management usually implies demanding surgeries and prolonged antibiotic regimens. In this Special Issue, Lucas et al. [2] evaluated the usefulness of multidisciplinary teams for this management. Through the evaluation of 86 cases where different options for the management of patients were analyzed, it was found that the management by multidisciplinary teams is the strongest predictor for success, especially when the DAIR procedure was used (*p* = 0.025). This analysis showed the benefit of these specialized teams given the complexity of these patients and the need for different treatment approaches to obtain the best outcome.

Another important issue in PJI management is obtaining the correct diagnosis. While conventional tools are still the reference methods for microbiological diagnosis [3] and methods of molecular biology have demonstrated complementary diagnostic value [4], there are still cases with negative microbiological results. The implications of biofilms in the pathogenesis of these infections are probably one of the causes of this problem. Schweizer et al. [5] performed an in vitro and experimental in vivo study that evaluated the usefulness of liquid saponin in sonication procedures. The use of saponin instead of saline increased the number of CFU recovered from 0.01 log_10_ to 2.2 log_10_ in an in vitro study using different bacterial species, and led to a >1 log_10_ increase in the recovery of *Staphylococcus epidermidis* in an in vivo experimental model. These data present the possibility of an increase in the sensitivity of microbiological culture-based studies.

The implications of infection etiology on patient outcomes are shown in two of the studies in this Special Issue. Over a period of 13 years, Auñon et al. [6] evaluated 499 patients diagnosed with PJI and compared monomicrobial infections with polymicrobial (18.2% of the cases) infections. Treatment success rates were significantly lower for polymicrobial infections, in both acute (*p* = 0.003) and chronic cases (*p* = 0.02). In another study, Liu et al. [7] analyzed the differences between *Pseudomonas aeruginosa* infection and other Gram-negative bacilli. In the study, 39 patients with *P. aeruginosa* were compared with 84 cases with other Gram-negative PJI, and the outcomes of both groups were similar, with no differences between them.

Treatment of PJI patients always requires a combination of surgery and antibiotics. A review by Tedeschi et al. [8] evaluated the use of fosfomycin as a broad-spectrum anti-biofilm agent that can be used against Gram-positive and Gram-negative organisms. Interestingly, fosfomycin is effective against the biofilms of methicillin-resistant *Staphylococcus* spp., vancomycin-resistant *Enterococcus* spp., carbapenem-resistant and extended-spectrum beta-lactamase-producing Enterobacterales, and MDR *P. aeruginosa*. Its clinical use (usually in combination with other antibiotics) showed the usefulness of this antibiotic in the management of difficult-to-treat osteoarticular infections.

Two articles analyze different surgical strategies for the management of osteoarticular infections. Mendoza Aguiló et al. [9] studied the implications of soft tissue reconstruction in the two-stage exchange of knee PJI. The analysis of 118 cases (40 with soft tissue reconstruction) showed that unreconstructed cases had worse outcomes than those with reconstruction (*p* = 0.029), suggesting that this strategy is an important tool in the multidisciplinary management of these patients. Salem et al. [10] studied the usefulness of vertical rectus abdominis myocutaneous flaps in the PJI setting. Despite the low number of cases, the good outcome indicates that this technique could be a useful method for wound management in cases of PJI.

Finally, the important issue of local treatment is studied by two articles. This type of treatment is important because it allows for increasing antibiotic levels in the infected tissues without facing the adverse effects of systemic treatment. The study by Coraça-Huber et al. [11] compares the release and antibacterial effectiveness of gentamicin eluted from Palacos^®^ R+G cement and tobramycin from Simplex^®^ T cement in an in vitro study. In the study, Palacos^®^ R+G exhibited higher and more sustained antibiotic release of gentamicin compared to tobramycin from Simplex^®^ T, with the former maintaining antibacterial activity for 42 days and the latter for 14 days. This release is important in the treatment of these biofilm-related infections, where higher antibiotic concentrations are needed during long periods to support bacterial eradication. Complementary to this study, Márquez-Gomez et al. [12] performed an observational study of clinical cases of PJI treated with new multiple antibiotic-loaded high-dose cement formulations and compared the outcome against classical antibiotic–PMMA cement combinations. Treatment failure was significantly lower for the new cement group compared to the classical approach (*p* = 0.005), with no differences in complications and with a shorter spacer cement time interval between stages. This demonstrates, again, the importance of the local treatment concept in the management of PJI.

In conclusion, the first edition of this Special Issue has shown that PJI continues to be a matter of ongoing discussion and research. The remaining open questions leave space and justification for further clinical studies with the goal of improving the management and treatment of these complex patients.

## Data Availability

No new data were created or analyzed in this study. Data sharing is not applicable to this article.

## Abbreviations

The following abbreviations are used in this manuscript:

PJI	Prosthetic Joint Infection
PMMA	Poly-Methyl-Methacrylate
MDR	Multidrug-Resistant
CFU	Colony-Forming Units
DAIR	Debridement, Antibiotics, and Implant Retention

## References

[1] Patel R. (2023). Periprosthetic Joint Infection. N. Engl. J. Med..

[2] Lucas J., Queirós J., Soares D., Carvalho A., Pereira F., Santos C., Sousa R., Abreu M.A. (2025). The Impact of Antibiotic Therapy Options and Multidisciplinary Approach in Prosthetic Joint Infections. Microorganisms.

[3] Esteban J., Patel R., Aguilera-Correa J.-J., Nelson S.B. (2025). Culture Working Group of the Unified PJI Definition Task Force Optimized Use and Performance of Culture for Periprosthetic Joint Infection Diagnosis: A Comprehensive Literature Review. Clin. Microbiol. Rev..

[4] Olearo F., Zein S.E., Portillo M.E., Zapf A., Rohde H., Berbari E.F., Wouthuyzen-Bakker M. (2025). Diagnostic Accuracy of 16S rDNA PCR, Multiplex PCR and Metagenomic next-Generation Sequencing in Periprosthetic Joint Infections: A Systematic Review and Meta-Analysis. Clin. Microbiol. Infect..

[5] Schweizer T.A., Egli A., Bosshard P.P., Achermann Y. (2025). Saponin Improves Recovery of Bacteria from Orthopaedic Implants for Enhanced Diagnosis Ex Vivo. Microorganisms.

[6] Auñón Á., Ortiz I., Peñarrubia S., Álvaro C., Torrecilla-Sádaba E., Garcia-Cañete J., Esteban J. (2025). Polymicrobial Prosthetic Joint Infections: Unraveling Risk Factors and Outcomes in a Single-Center Study. Microorganisms.

[7] Liu W.-Y., Hendriks J.G.E., van Kempen R.W.T.M., van der Weegen W., Rijnen W.H.C., Goosen J.H.M., van der Zwaard B.C., Pronk Y., Zijlstra W.P., Ten Have B.L.E.F. (2025). Clinical Outcome of Patients with Acute Periprosthetic Joint Infections Caused by *Pseudomonas aeruginosa* Compared to Other Gram-Negative Bacilli. Microorganisms.

[8] Tedeschi S., Giannitsioti E., Mayer C. (2025). Emerging Concepts for the Treatment of Biofilm-Associated Bone and Joint Infections with IV Fosfomycin: A Literature Review. Microorganisms.

[9] Mendoza Aguiló C., Vicente M., Cano A., López Martínez J.A., Bulla A., Amat C., Serracanta J., Corona P.S. (2026). Soft Tissue Reconstruction Does Not Compromise Infection Control in Chronic Knee Periprosthetic Joint Infection Treated with Two-Stage Exchange Arthroplasty Despite Increasing Complexity. Microorganisms.

[10] Salem O., Zhang J., Grammatopoulos G., Garceau S., Abdelbary H. (2025). A Single Tertiary-Care Center Case Series Using Vertical Rectus Abdominis Myocutaneous Flap in the Management of Complex Periprosthetic Joint Infection of the Hip. Microorganisms.

[11] Coraça-Huber D., Humez M., Kühn K.-D. (2025). A Comparative Study of Extended Gentamicin and Tobramycin Release and Antibacterial Efficacy from Palacos and Simplex Acrylic Cements. Microorganisms.

[12] Márquez-Gómez M., Prats-Peinado L., Díaz J.A.M., Sánchez-Somolinos M., Guembe M., Vaquero J., Sanz-Ruiz P. (2026). Clinical Efficacy and Safety of Multiple High-Dose Antibiotic-Loaded Cement Spacers in the Two-Stage Revision of Gram-Positive Periprosthetic Joint Infection. Microorganisms.

